# Resistance Genes, Phage Types and Pulsed Field Gel Electrophoresis Pulsotypes in *Salmonella* *enterica* Strains from Laying Hen Farms in Southern Italy

**DOI:** 10.3390/ijerph10083347

**Published:** 2013-08-06

**Authors:** Antonio Camarda, Nicola Pugliese, Antonia Pupillo, Marta Oliva, Elena Circella, Anna Maria Dionisi, Antonia Ricci, Marilisa Legretto, Anna Caroli, Carlo Pazzani

**Affiliations:** 1 Department of Veterinary Medicine, Università di Bari, strada provinciale per Casamassima Km 3, Valenzano-Bari 70010, Italy; E-Mails: antonio.camarda@uniba.it (A.C.); nicola.pugliese@uniba.it (N.P.); elena.circella@uniba.it (E.C.); marilisalegretto@libero.it (M.L.); anna.caroli@tiscali.it (A.C.); 2 Department of Biology, Università di Bari “Aldo Moro”, Via E. Orabona, 4 70125 Bari, Italy; E-Mails: antonellapupillo@libero.it (A.P.); marta.oliva@uniba.it (M.O.); 3 Department of Infectious, Parasitic and Immuno-Mediated Diseases, Istituto Superiore di Sanità, Rome 00161, Italy; E-Mail: annamaria.dionisi@iss.it; 4 National Reference Laboratory for Salmonella, Istituto Zooprofilattico delle Venezie, Viale dell’Università 10, 35020 Legnaro (Padova), Italy; E-Mail: aricci@izsvenezie.it

**Keywords:** IncN, IS*26*, phage types, resistance genes, *Salmonella enterica*, *tet*(C)

## Abstract

Twenty-four *Salmonella* *enterica* isolates (13 serovar Enteritidis and 11 Typhimurium) isolated from 5,600 samples from intensive laying hen farms in Italy in 1998–2007 were characterized for antimicrobial resistance genes, pulsotype and phage type. Most of *S.* Typhimurium strains were pulsotype STYMXB.0147 (81.8%), phage type DT143 and resistant to sulfamethoxazole encoded by *sul2*. Two multidrug resistant (MDR) strains were identified. One strain, STYMXB.0061, was resistant to ampicillin (A), chloramphenicol (C), streptomycin (S), sulfamethoxazole (Su) and tetracycline (T) encoded by the Salmonella Genomic Island SGI1. The second MDR strain, STYMXB.0110, was resistant to SSuT encoded by *sul1* and *sul2*, *aadA1* and *tet*(C)-flanked by an IS26 element, respectively. The *tet*(C) gene has been reported to confer low levels of resistance and it has very rarely been detected in *S*. Typhimurium from poultry. In the current study, the MIC value (32 µg/mL) was consistent with the breakpoint (≥16 µg/mL) reported for *Enterobacteriaceae*. Most of the *S*. Enteritidis strains were resistant to Su (encoded by *sul2*). One MDR strain (ANxSSuT) was identified. With the exception of nalidixic acid (Nx), the resistances were respectively encoded by *bla*_TEM_, *strAB*, *sul2* and *tet*(A) harbored by an IncN conjugative plasmid. All isolates were pulsotype SENTXB.0001 with PT14b being the most prevalent identified phage type (57.1%). In Europe, SENTXB.0001 is the predominant PFGE profile from clinical cases and the identification of PT14b has steadily been on the increase since 2001. The findings presented in this study highlight the potential spread of *S*. Enteritidis phage types PT14b and *S*. Typhimurium DT143 in a field of particular relevance for zoonoses. Additional, the presence of resistance genes and genetic elements (conjugative plasmid and IS element) underlines the need to assess routinely studies in field, such as poultry farms, relevant fot the public health and suitable for the storage and diffusion of antimicrobial resistance.

## 1. Introduction

*Salmonella enterica* is a leading cause of zoonotic food-borne infections and a concern for public health. *S. enterica* subspecies *enterica* serovars Enteritidis and Typhimurium (hereafter referred to as *S*. Enteritidis and *S.* Typhimurium, respectively) are frequently isolated from both human and animal infections [1]. Human salmonellosis is mainly associated with the consumption of poultry-derived products (meat and eggs) [2]. In Europe and the USA, the most commonly-associated serovar is *S*. Enteritidis [3,4]. This is probably linked to its ability to colonize the reproductive organs of hens and thus to contaminate eggs [5,6].

The emergence of antimicrobial resistance among *S*. Enteritidis and *S*. Typhimurium strains makes it more difficult to choose the proper antimicrobial therapy, when required [7]. Particularly noteworthy is the global spread of *S*. Typhimurium phage type DT104 ACSSuT which is resistant to ampicillin (A), chloramphenicol (C), streptomycin (S), sulphonamides (Su), and tetracycline (T) and is currently one of the most prevalent penta-resistant serovars isolated from animals [8]. This multidrug resistance is encoded by the Salmonella Genomic Island (SGI1) [9,10]. Within the 43 kbp SGI1 element lies a 13 kbp region harbouring the *aadA2*, *floR*, *tet*(G), *bla*_PSE-1_, *sul1* gene cluster encoding resistance for streptomycin, chloramphenicol, tetracycline, ampicillin and sulfamethoxazole, respectively. Additional to S. Typhimurium DT104, it is important to underline the recent worldwide diffusion of the emerging *S*. enterica subspecies serovar 4,[5],12:i:– (S. 4,[5],12:i:–), defined as a monophasic variant of *Salmonella* Typhimurium. In Europe monophasic variant strains are often characterized by antimicrobial resistance to ASSuT encoded by the *bla*_TEM_, *strA-strB*, *sul2* and *tet*(B) genes, respectively [11].

The constant monitoring of antimicrobial resistance among those bacteria which play a significant role in veterinary and human medicine is therefore of great importance. On this matter the European Food Safety Authority has highlighted the need to perform genetic characterization of antimicrobial resistances [12]. If genetic characterization of antimicrobial resistances were combined with molecular strain typing, it would greatly benefit knowledge on the spread of resistance genes among the clonal groups of *Salmonella* strains principally identified in the veterinary field and potentially involved in zoonoses. Of the different typing techniques available, Pulsed-Field Gel Electrophoresis (PFGE) has become universally-recognized as the reference method for molecular subtyping [13]. Indeed, PFGE profiles can be compared with those included in the PulseNet-Europe international database and assigned to specific pulsotypes. Data available and currently accumulated on the identified pulsotypes represent valuable information for molecular-epidemiology studies.

In the current study the genetic basis of the antimicrobial resistance and the clonal relatedness among *S.* Enteritidis and *S.* Typhimurium isolates isolated from intensive laying hen farms in southern Italy in 1998–2007 was determined. The study covers retrospectively the period before the implementation of a compulsory national program for the control of *S.* Enteritidis and *S.* Typhimurium in laying hens in Italy (approved by the Health Department on February 2008,) following the European Union regulation no. 2160/2003.

## 2. Methods

### 2.1. Samples and Bacterial Strains

Between 1998 and 2007 a total of 5,600 samples from 560 inspections were collected from 16 intensive laying hen farms in southern Italy, ranging from 10,000 to 100,000 hens per farm, with a median of 28,500. Animals were reared in traditional cages in all farms. The prophylaxis program of the farms did not included vaccination against *S.* Enteritidis and *S.* Typhimurium, but an attenuated live vaccine was administered against *Salmonella enterica* subsp. *enterica* ser. Gallinarum.

During the ten year period, inspections were conducted every 15 weeks. Each inspection is intended for one farm and all the 16 farms were tested each year. From each farm, the samples were collected as follows: two from feces, eggs (30 eggs per sample) and shed dust; one from feedstuff, selection room floor, egg graders and worker’s shoes. The process of identification of *S*. *enterica* per sample was in accordance with the procedure described in ISO6579:1993 for samples collected until 2002. Samples collected from 2003 to 2007 were processed following the procedure described in ISO6579:2002 [14]. At least five suspected colonies were then identificated and serotyped per positive sample. Serotyping was performed by slide agglutination using commercially available sera (Bio Rad, Milan, Italy) according to the White-Kauffmann-Le Minor scheme [15]. The European legislation in force during the sampling period (in particular the Council Directive 92/117/EEC) provided the notification for *Salmonella* only limited to the serovars Enteritidis and Typhimurium, not considering other serovars as zoonotic agents. In accordance with this legislation, the microbiological analysis was focused on detection of *S*. Enteritidis and *S.* Typhimurium strains. Detection of *S*. enterica subspecies serovar 4,[5],12:i:- (S. 4,[5],12:i:-), defined as a monophasic variant of *Salmonella* Typhimurium, was also included in this study.

All isolated *S.* Enteritidis and *S.* Typhimurium strains were preliminary characterized by phage typing and antimicrobial susceptibility. When two or more isolates from the same inspection exhibited the same phage type and antimicrobial susceptibility pattern, only one was chosen as representative and included in further studies.

### 2.2. Clonality—PFGE and Phage Typing

Clonal relationship was assessed by PFGE and established according to criteria described by Tenover *et al.* [16]. Genomic restriction was performed according to the standardized *Salmonella* protocol of the CDC PulseNet [17]. Agarose-embedded DNA was digested with 40 U of *Xba*I for 3 h at 37 °C. The restriction fragments were separated by electrophoresis in Tris-borate-EDTA (44,5 mM Tris-borate, 1 mM EDTA; pH 8,0) at 14 °C for 20 h using a CHEF-DR III (Bio-Rad, Milan, Italy). Electrophoresis conditions were as follows: 6 V/cm, angle of 120 °C, for 20 h with pulse times of 2.2 to 63.8 s. The *Salmonella* Braenderup H9812 strain was used as a molecular standard. The PFGE agarose gels were stained with ethidium bromide (40 µg/mL) and the DNA band images were acquired by the Gel Doc-It photo documentation system (Gel Doc-It photo documentation system, UVP, Upland, CA, USA). 

Digital images of the PFGE profiles were analyzed using algorithms available in the BioNumerics software package (Applied Maths, Sint-Martens-Latem, Belgium). DNA profiles differing in one or more DNA fragments were considered distinct patterns. Strains with a coefficient of similarity ≥ 90% were considered as genetically closely related. All PFGE profiles were compared with those included in the PulseNet-Europe international database and named with a six letter code followed by a four digit numerical identifier, for example: STYMXB.0006 [18]. Phage typing was performed according to the standard procedure [19].

### 2.3. Antimicrobial Susceptibility Testing

Antimicrobial susceptibility testing was carried out by the agar disk diffusion test on Mueller-Hinton agar (Oxoid, Milan, Italy), following the Clinical and Laboratory Standards Institute (CLSI) guidelines [20]. The antimicrobial disks were: ampicillin (A; 10 µg), chloramphenicol (C; 30 µg), gentamicin (CN; 10 µg), kanamycin (K; 30 µg), nalidixic acid (Nx; 30 µg), streptomycin (S; 10 µg), sulfamethoxazole (Su; 25 µg), tetracycline (T; 30 µg) and trimethoprim (W; 5 µg). The Minimal Inhibitory Concentration (MIC) for tetracycline (strains ST256) was determined using the macrodilution (tube) broth method as described by the Clinical and Laboratory Standards Institute (CLSI) guidelines [21]. *E. coli* ATCC 25922 was used as a quality control strain.

Bacteria non susceptible to at least one agent in three or more antimicrobial categories we tested (namely aminoglycosides, folate pathway inhibitors, penicillins, phenicols and tetracyclines) were defined as multidrug-resistant (MDR) [22].

### 2.4. Antimicrobial Resistance Genes/Genetic Elements and Conjugation Assays

Primers for PCR detection of SGI1, class 1 integrons, TnA family transposons, IS*26* element and the antimicrobial resistance genes *aadA1*, *aadA2* and *strAB* (antimicrobial category aminoglycosides), *bla*_PSE-1_ and *bla*_TEM _ (penicillins), *floR* (phenicols), *sul1* and *sul2* (folate pathway inhibitors), *tet*(B), *tet*(C) and *tet*(G) (tetracyclines) were as reported previously [23] or listed in Table S1 (see Online Supplementary Information, Table S1 for primers used in this study). The primers designed for the current study were based on sequences available in GenBank using the Primer3 (version 4.0.0) software (http://primer3.wi.mit.edu). Genomic DNA was extracted as previously described [24]. PCRs were performed in a total volume of 25 µL containing 50 to100 ng of total DNA, 1X PCR buffer (10 mM Tris-HCl, 50 mM KCl, 1.5 mM MgCl_2_; pH8.3), 200 µM of each deoxynucleoside triphosphate (dNTP), 20 µM of each primer and 1 U *Taq* polymerase (Takara Bio Inc., Otsu, Shiga, Japan).

Antimicrobial resistance gene cassettes integrated into class 1 integrons were amplified with primers 5CS-F and 3CS-R (Table S1) and cloned into a commercial vector (Promega, Milan, Italy) in accordance with the manufacturer’s instructions. *E. coli* JM109 was used as a recipient strain. The cloned products were purified before sequencing using a commercial kit (Promega) and sequenced by the BMR Genomics (Sequencing service, BMR Genomics, Padova, Italy). The resulting DNA sequences were analyzed for similarity by using the BLAST program available on the NCBI BLAST homepage [25].

Cloning of tet(C) from strain 256 was performed by *Sau*3AI (Takara Bio Inc.) partial restriction of genomic DNA. Restriction fragments were separated through an agarose gel (1% w/v) in 40 mM Tris-Acetate, 1 mM EDTA buffer, at 5 V/cm to the gel and fragments ranging from 1 to 4 kb were purified with a commercial Kit. Plasmid pBluescript II SK(-), encoding resistance to ampicillin, was digested with *Bam*HI and dephosphorylated (New England Biolabs, Ipswich, MA, USA). Ligations were performed at 16 °C for 16 h and competent cells of *E. coli* JM109 were transformed with the recombinant plasmids and plated on Luria Bertani (LB) agar plates supplemented with tetracycline (Sigma Aldrich, Milan, Italy), (final concentration of 10 µg/mL). Following incubation at 37 °C for 16 h the candidate colonies were purified by single-colony isolation and grown on LB agar plates supplemented with tetracycline and ampicillin (final concentration of 10 and 100 µg/mL, respectively). Plasmids were isolated from each candidate, purified and reintroduced into JM109 competent cells to confirm their encoding resistance to tetracycline. All purified plasmids were characterized by restriction maps and one candidate plasmid, named pBA97, was chosen for its largest DNA insert (2,688 bp). The DNA sequence was determined and submitted to GenBank (accession number GU987054).

Conjugation experiments were performed at 37 °C as described previously [24]. Matings were also performed at 25 °C to detect any thermosensitive transfer of plasmids such as those of the IncH1 group [26]. Antimicrobials on plates were: A (100 µg/mL), C (25 µg/mL), S (100 µg/mL) and T (10 µg/mL and 20 µg/mL) for selection of antimicrobial resistance trasferred by conjugation. These antimicrobials were singularly added to Nx (50 µg/mL) or rifampicin-RD (100 µg/mL) for selection of transconjugants from the mixuture of conjugation. 

*E. coli* K-12 strain ZM46, a nalidixic acid-resistant mutant of CSH26, or a rifampicin mutant of *E. coli* K-12 were used as recipient strain. The frequency of transfer of a genetic marker was expressed as the number of transconjugants per donor cell. Plasmids were typed by the PCR Based Replicon Typing protocol (PBRT) using positive controls kindly supplied by A. Carattoli [27].

## 3. Results and Discussion

### 3.1. Salmonella Prevalence in Samples and Farms

*S.* Enteritidis or *S.* Typhimurium strains were isolated from 39 out of the 5,600 collected samples (see Online Supplementary Information, Table S2 for distribution of S. Enteritidis and S. Typhimurium strains isolated from laying hen farms in 1998-2007). There were no monophasic variants among the *S.* Typhimurium isolates. Most of the positive samples were feces and eggshell (17 and 13, respectively). No feedstuff or dust sample resulted positive. The low ratio of positive samples did not allow us to perform a statistic comparison of the *S.* Enteritidis/*S*. Typhimurium prevalence among samples. During the ten-year period, all farms resulted positive at least once (Table S2). In 13 farms the isolation of *S.* Enteritidis/*S*. Typhimurium was sporadic; while three farms (namely C, E and L) were positive more than one time. Only in one farm (L) was *S.* Enteritidis detected following two consecutive inspections.

Twenty-four strains of *S. enterica* (13 serovar Enteritidis and 11 serovar Typhimurium) were included for further characterization (Table 1).

### 3.2. Clonality Study

**Figure 1:**
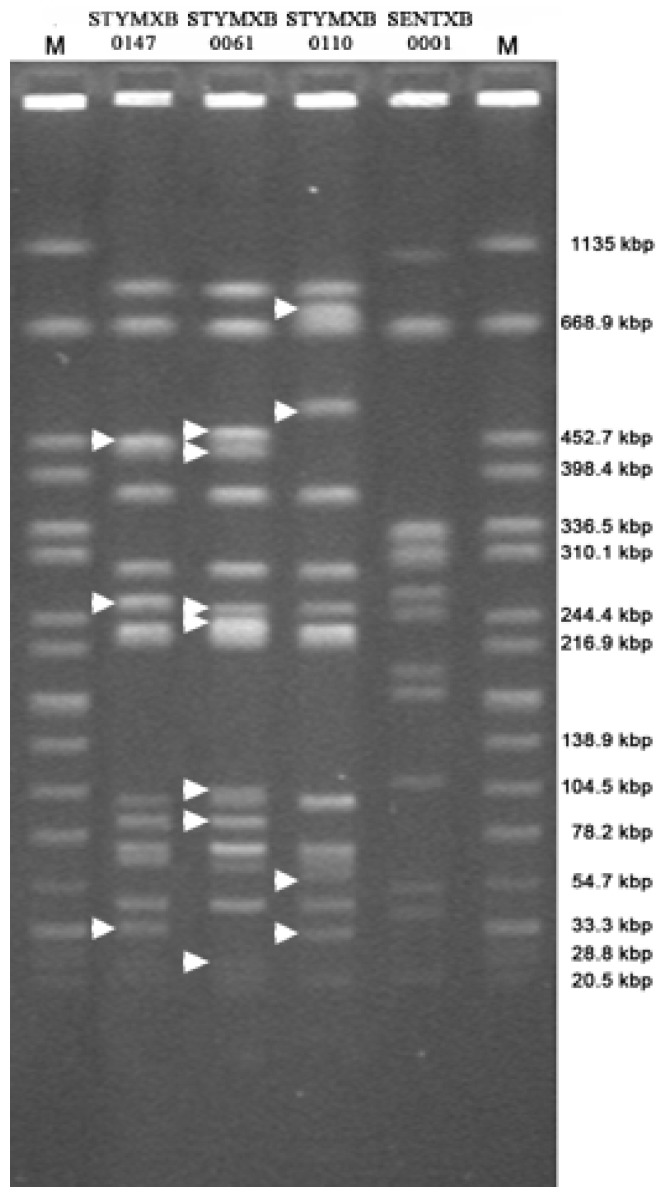
Pulsed field gel electrophoresis profiles exhibited by the *Salmonella* *enterica* strains analyzed in the current study. All representative profiles are included. Lines 1 and 6: universal standard, *Salmonella* serotype Braenderup H9812. Line 2: *S.* Typhimurium strain(s) R-type ACSSuT (pulsotype STYMXB.0061). Line 3: *S.* Typhimurium strain ST256 (R-type NxSSuT and pulsotype STYMXB.0110). Line 4: *S.* Typhimurium strain(s) pulsotype STYMXB.0147. Line 5: *S.* Enteritidis strain(s) pulsotype SENXB.0001. White arrows highlight restriction fragments distinguishing the pulsotypes STYMXB.

Clonal relatedness was established by analysis of PFGE profiles (Figure 1) which were first compared with profiles included in the PulseNet-Europe international database. All the *S*. Enteritidis strains exhibited an indistinguishable pattern which was 95% identical to the pulsotype SENTXB.0001. This pulsotype has been reported as the predominant PFGE profile exhibited by clinical *S*. Enteritidis strains isolated in Europe [28].

Three PFGE profiles were detected in *S*. Typhimurium strains. Most of the isolates (81.8%) exhibited a pattern 100% identical to the PFGE profile of the pulsotype STYMXB.0147. The PFGE profiles exhibited by the remaining strains namely ST425 and ST256 were assigned to the pulsotypes STYMXB.0061 (100% identity) and STYMXB.0110 (96.3% identity), respectively. The three identified pulsotypes differed from each other by 7 to 11 restriction fragments. According to the criteria for interpreting PFGE patterns reported by Tenover *et al.* [16], the strains of one pulsotype (e.g., STYMXB.0147) were classified as unrelated to strains belonging to the other two pulsotypes (e.g., STYMXB.0061 or STYMXB.0110). The pulsotype STYMXB.0061 is one of the most common profiles identified in *S*. Typhimurium and in Italy it was the second most common PFGE profile identified in human isolates during 2003–2006 [11,29]. To the authors’ knowledge, no epidemiological data are available for the pulsotypes STYMXB.0147 and STYMXB.0110 and further investigations are needed to assess their role both in different animal species and in humans.

Five different phage types were identified within the *S*. Enteritidis strains analyzed in this study with phage type PT14b being the most prevalent and detected in 16 out of the 28 *S*. Enteritidis isolates (57.1%) (Table S2). Before 2001, *S*. Enteritidis PT14b was rarely considered a cause of human salmonellosis in Europe [30]. However, since 2001, the isolation of *S*. Enteritidis PT14b from clinical cases has been increasing with an upsurge in nalidixic acid resistance in late 2010 [31,32]. In Europe phage type PT14b is mainly associated with pulsotype SENTXB.0002 and less frequently with SENTXB.0001 [28]. However, the linkage between PT14b and SENTXB.0001 or SENTXB.0002 also depends on the place of isolation.

For instance, in Italy PT14b is strongly associated with SENTXB.0001 but little information is available on the extent of their presence and/or distribution in animals such as poultry. Despite the low number of isolates, the findings presented in the current study have shed light on the possible role played by intensive laying hen farms both as a reservoir and propagator of the main PT14b-SENTXB.0001 *S*. Enteritidis clinical group of strains and its potential concern for public health.

**Table 1:** Antimicrobial susceptibility, phage types, pulsotypes, PCR detection of SGI1, class 1 integrons and resistance genes in *S. enterica* strains isolated from laying hen farms in Italy in 1998–2007.

PulseNet Europe	Serovar	Phage type	Place/Year	Farm	Sample	Resistance	Class 1 integrons			SGI1 ^‡^	
nomenclature	(n. of strains)	(n. of strains) ^†^				pattern (strain) *					
							*intI1*	gene	Resistance	left	right
								cassette(s)	gene (s)	junction	junction
SENTXB.0001	Enteritidis (2)	PT1 (1)	Apulia/1998	A	Workers’ shoes	Su	-	*-*	*sul2*	-	-
		PT37 (1)	“	B	Feces	"	-	*-*	*"*	-	-
SENTXB.0001	Enteritidis (1)	PT8 (1)	Apulia/2001	I	Eggs	"	-	*-*	*"*	-	-
SENTXB.0001	Enteritidis (2)	PT4 (1)	Apulia/2003	J	Feces	"	-	*-*	*"*	-	-
		PT14b (1)	“	K	Eggs	"	-	*-*	*"*	-	-
SENTXB.0001	Enteritidis (4)	PT14b (2)	Apulia/2004	L	Feces; eggs	"	-	*-*	*"*	-	-
		PT14b (1); NT (1)	“	C	Feces Eggs	"	-	*-*	*"*	-	-
SENTXB.0001	Enteritidis (1)	NT (1)	Apulia/2005	M	Feces	"	-	*-*	*"*	-	-
SENTXB.0001	Enteritidis (2)	PT14b (1)	Apulia/2006	L	Feces	"	-	*-*	*"*	-	-
		NT (1)		N	Egg grader	"	-	*-*	*"*	-	-
SENTXB.0001	Enteritidis (1)	NT (1)	"	O	Eggs	ANxSSuT (SE402)	-	*-*	*bla*_TEM-1_*; strAB; sul2; tet*(A)	-	-
STYMXB.0147	Typhimurium (3)	DT193 (2)	Apulia/1999	C	Eggs;eggs grader	Su	-	*-*	*sul2*	-	-
		NT (1)	“	C	Eggs	"	-	*-*	*"*	-	-
STYMXB.0147	Typhimurium (4)	DT193 (1)	Apulia/2000	C	Feces	"	-	*-*	*"*	-	-
		DT193 (1)	"	F	Feces	"	-	*-*	*"*	-	-
		DT104 (1)	"	G	Egg grader	"	-	*-*	*"*	-	-
		U302 (1)	"	H	Selection room floor	"	-	*-*	*"*	-	-
STYMXB.0147	Typhimurium (1)	DT104 (1)	Apulia/2006	E	Feces	"	-	*-*	*"*	-	-
STYMXB.0147	Typhimurium (1)	DT193 (1)	Basilicata/1999	D	Eggs	"	-	*-*	*"*	-	-
STYMXB.0110	Typhimurium (1)	DT208 (1)	Apulia/2000	E	Feces	SSuT (ST256)	+	*aadA1*	*sul1; sul2; tet*(C)	-	-
STYMXB.0061	Typhimurium (1)	DT104 (1)	Basilicata/2007	P	Feces	ACSSuT (ST425)	+	*aadA2; bla*_PSE-1_	*sul1; floR; tet(*G)	+	+

* A, ampicillin; C, chloramphenicol; Nx, nalidixic acid; S, streptomycin; Su, sulfamethoxazole; T, tetracycline; ^†^ NT: not typeable; ^‡^ Key: +, positive; -, negative.

Among the *S*. Typhimurium strains DT193 was the prevailing phage type (45.4%). All isolates DT193 were STYMXB.0147 and, as previously mentioned, no epidemiological data are available on this pulsotype. No *S*. Typhimurium strain STYMXB.0147 was identified in a survey conducted on clinical isolates from nine European countries in 2000–2004 [33]. However, DT193 *S*. Typhimurium strains (as well as DT204, DT204c and DT29) were responsible for human gastroenteritis-outbreaks (with bovine reservoirs) in the United Kingdom in the 1970s and DT193 is one of the most common long term phage types identified from clinical isolates in Slovakia [34,35,36]. DT193 is also a phage type of great concern in Brazil where its detection amongst clinical isolates in *S*. Typhimurium isolates has been increasing since the 1990s [37]. Unfortunately, no pulsotype is available for the DT193 *S*. Typhimurium strains described in those studies. Such data would have been valuable in assessing the levels of diffusion of *S*. Typhimurium STYMXB.0147, DT193 strains among clinical isolates.

### 3.3. Antimicrobial Susceptibility Study

Among the 24 *S. enterica* isolates included in the present study three were multidrug-resistant (MDR). One *S.* Enteritidis strain (named SE402) exhibited resistance to ampicillin, nalidixic acid, streptomycin, sulfamethoxazole and tetracycline (ANxSSuT R-type). Of the two MDR *S.* Typhimurium strains ST425 was resistant to ampicillin, chloramphenicol, streptomycin, sulfamethoxazole and tetracycline (R-type ACSSuT) while ST256 exhibited resistance to streptomycin, sulfamethoxazole and tetracycline (R-type SSuT). Regardless of the serovar, the remaining isolates only exhibited resistance to sulfamethoxazole (R-type Su).

### 3.4. Genetic Elements and Resistance Genes

The incidence of class1 integrons in S. Typhimurium strains analyzed in this study was 18.2%. Strains ST425 (R-type ACSSuT) and ST256 (R-type SSuT) were PCR positive for *intI*1, yielding an amplicon of the expected molecular size of 838 bp. ST425 was also PCR positive for the left and right junctions of SGI1, yielding the expected amplicons of 500 and 515 bp, respectively.

Gene cassettes integrated in class 1 integrons were detected by the primers 5CS-F and 3CS-R which specifically anneal to the 5’ and 3’ conserved regions of class 1 integrons, respectively. One amplicon (accession number GU987053) was detected in *S*. Typhimurium strain ST256. The DNA sequence was determined and an Open Reading Frame (ORF) of 852 bp was found to be homologous with the *aadA1* gene which encodes resistance for streptomycin. Two amplicons (accession numbers GU987052 and GU987051) were obtained from the strain ST425: their nucleotide sequence was determined and two ORFs of 852 bp and 987 bp were identified, respectively. The ORF of 852 bp was classified as *aadA2* which encodes resistance for streptomycin. The ORF of 987 bp was classified as *bla*_PSE-1_, which encodes resistance for ampicillin. PCR analysis was further extended for the detection of *sul1* and its linkage to class 1 integrons. *sul1* was found associated just with the class 1 integrons harboring the *aadA1* and *bla*_PSE-1_ gene cassettes.

Analysis of antimicrobial resistance genes was completed by PCR detection of the *bla*_TEM_, *floR*, *strAB*, *sul2*, and *tet*(A), *tet*(B), *tet*(G) determinants which are commonly found in MDR *Salmonella* strains and confer resistance to ampicillin, chloramphenicol, streptomycin, sulfamethoxazole and tetracycline, respectively [38,39,40].

**Figure 2:**
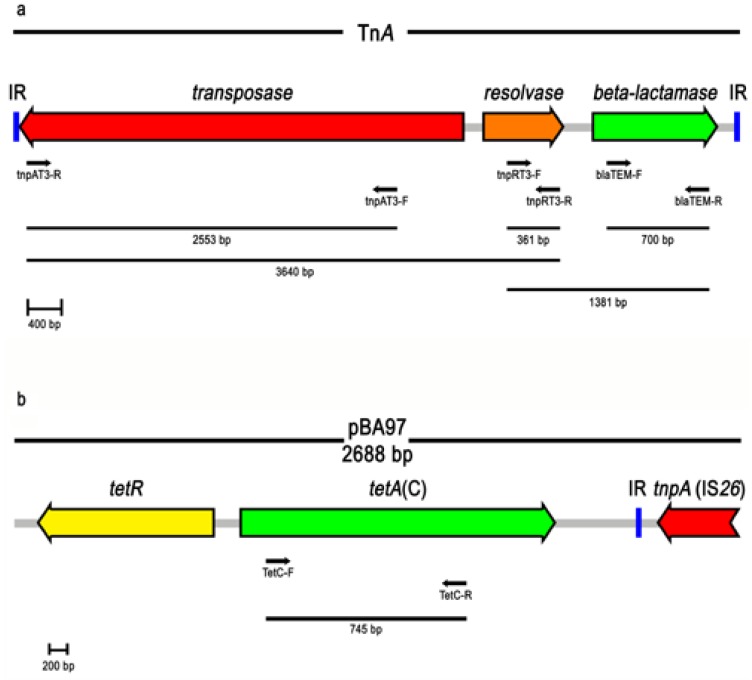
Schematic representation of both TnA family transposon (**a**) and pBA97 construct (**b**) (GenBank accession numbers V00613 and GU987054, respectively). Direction of transcription is indicated by thick arrows. The primers and their directions are represented by narrow arrows. The horizontal bars and their sizes represent the DNA fragments yielded by different primer combinations.

*S.* Enteritidis strain SE402 was found PCR positive for *bla*_TEM_, *strAB*, *sul2* and *tet*(A). The *bla*_TEM_ gene has also been found in a group of closely related transposons: namely Tn*1*, Tn*2* and Tn*3* (TnA family transposons) which are three of the earliest bacterial resistance transposons to have been identified [41]. Apart from *bla*_TEM_, the transposon TnA family contains the *tnpA* (transposase) and *tnpR* (resolvase) genes, as well as the resolvase site *res* [42]. The *bla*_TEM_ gene identified in SE402 was therefore analyzed to verify if it was part of a TnA-like element. PCR assays with primers specific for *tnpA* and *tnpR* gave the two expected amplicons of 2543 bp and 361 bp, respectively. The *bla*_TEM_-*tnpR*–*tnpA* gene array was also established by PCR validating the presence of a putative TnA-like element (Figure 2a). In *E*. *coli* recovered from poultry meat *sul2* has been found associated with *strAB* [43]. PCR was used to demonstrate that this was also the case for the *sul2* and *strAB* genes identified in SE402. An attempt to detect if the identified TnA-like tranposon were close to the *sul2*-*strAB* cluster failed.

Apart from *aadA2*, *bla*_PSE-1_ and *sul1* the strain ST425 was positive for PCR detection of *floR* and *tet*(G) genes. The *aadA2*, *floR*, *tet*(G), *bla*_PSE-1_, *sul1* genes were found, by PCR, organized as a gene array and proved indistinguishable to that reported for the genetic element SGI1 which has been frequently detected in *S*. Typhimurium strains DT104 and R-type ACSSuT [9]. Interestingly, indistinguishable molecular (pulsotype STYMXB.0061), genetic (*aadA2*, *floR*, *tet*(G), *bla*_PSE-1_ and *sul1* gene cluster) and phenotypic (R-type ACSSuT, phage type DT104 or the related DT120 and U302 types) features exhibited by ST425 were also found in *S*. Typhimurium strains isolated from rabbit farms in the same geographic areas [23]. Although these findings might be independent of each other, a possible cross-contamination (e.g., mediated by humans or movement of eggs or farm equipment) cannot be excluded.

The *S*. Typhimurium strain ST256 (R-type SSuT) which was positive for *aadA1* and *sul1* was also found positive for *sul2* but negative for *tet*(A), *tet*(B) and *tet*(G). The genetic basis of tetracycline resistance was then determined by cloning a 2,688 bp *Sau*3AI DNA fragment into pBluescript II SK(-). The resulting construct, named pBA97, enabled JM109 cells to grow on LB agar plates supplemented with tetracycline. The DNA sequence was determined and two ORFs of 1173 (ORF1) and 657 bp (ORF2) were identified and found to be homologous with the *tet*(C) and *tet*R genes, respectively (Figure 2b). The DNA sequence also included a portion of an IS*26* element that lay beside *tet*(C). The presence and linkage between *tet*(C) and the putative IS*26* element was then confirmed by PCR. An attempt to detect *tet*(C) organised within a composite transposon failed. No IS*26* element next to *tet*R was identified (PCR conditions for long amplifications up to 20 kb).

Tetracycline is a broad-spectrum agent widely used for bacterial infections in human and veterinary medicine [44]. The most frequently found types of *tet* genes detected in *Salmonella* belong to the Group-I with *tet*(A), *tet*(B) and *tet*(G) being those commonly reported while *tet*(C) and *tet*(D) are more rarely found [38,45]. In *S*. Typhimurium *tet*(C) has been detected in strains isolated both from clinical cases and animal sources [46,47]. Additionally, *tet*(C) has also been described to confer low levels of resistance [48]. In *E*. *coli* strains isolated from various animal and environmental sources the reported MIC values ranged between 2 and 16 µg/mL (intermediate susceptibility). However, to the best of the authors’ knowledge only in one study has *tet*(C) been reported in *S*. Typhimurium isolated from poultry and no MIC value was specified [8]. In ST256 the MIC was found to have a value of 32 µg/mL which is consistent with the breakpoint (≥16 µg/mL) reported for *Enterobacteriaceae* [49]. It is interesting to note that an IS*26* element flanking *tet*(C) was detected. IS*26* associated with antibiotic resistance genes has been assumed to be involved in their dissemination [50]. In the light of this hypothesis the findings given in this paper raise concern on the possible role played by IS*26* in the diffusion of *tet*(C) among *S*. Typhimurium strains detected in a field of particular relevance for zoonoses. 

The strains which were only resistant to sulfamethoxazole were all found PCR positive for *sul2*. Dissemination of this gene has also been reported and further investigations are needed to assess its wide diffusion [8,43,51].

To ascertain whether the multi-resistance was encoded by genes located on self-transmissible genetic elements conjugation experiments were performed between MDR *Salmonella* isolates and *E. coli* recipient strains. Resistances exhibited by the strain SE402 (with the exception of that to nalidixic acid) were transferred as a linkage group to *E. coli* with an average frequency of about 1 × 10^−8^ transconjugants per donor cell. No determinant of resistance was transferred in matings between the strains ST256 or ST425 and *E. coli* (detection frequency less than 1 × 10^−9^). Strain SE402 harboured two distinctive groups of plasmids: IncFII and IncN. The latter was also detected in *E. coli* transconjugants, demonstrating that the resistance to ASSuT was encoded by genes harbored by an IncN plasmid whose incompatibility group has been reported to have a broad host spectrum [52]. Plasmids of the group IncFII and IncI1 were detected in strain ST256. However, no *E. coli* transconjugants were identified in matings with ST256. This excluded the possibility that resistance exhibited by ST256 might be mediated by transferable plasmids. 

In *S*. Enteritidis of clinical origin conjugative IncN plasmids play a key role in the dissemination of a range of antimicrobial resistances, principally ampicillin, streptomycin and tetracycline [53,54]. The incompatibility group IncN has also recently been identified in an *S*. Enteritidis strain isolated from poultry [55]. However, it was not specified whether the IncN plasmid carried antimicrobial resistance genes or if it was conjugative. The findings presented in the current study have highlighted the presence of this group of plasmids among the widely-detected Enteritidis serotype [56].

## 4. Conclusions

Surveillance of antimicrobial resistance exhibited by *Salmonella* isolates of animal origin addresses fundamental epidemiological issues such as the spread of MDR strains, the diffusion of antimicrobial resistance genes and their prevalence among the most commonly detected clones. Of particular relevance for public health is the assessment of the extent of those clones commonly detected among clinical cases and animal sources. Not least is also the identification of new clones present among intensive animal farms and potentially transmissible to humans. This study also provides retrospective information about antimicrobial resistance and the related genetic elements of *Salmonella* strains isolated before the implementation of a compulsory national program of monitoring.

It is important to highlight that *S*. *enterica* infections in chicken are often asymptomatic [57]. Poultry farms may thus be an insidious environment for the storage and diffusion of antimicrobial resistance genes and genetic elements. In the ten years period of survey, all farms included in this study were positive for isolation of *S*. Enteriditis and/or *S*. Typhimurium at least once. Additionally, in the three detected MDR isolates most of the identified resistance genes were linked to mobile genetic elements. Of particular relevance is also the potential spread of both resistance genes and associated genetic elements among those phage types and pulsotypes which are potentially harmful to human health. The results presented here reinforced the need to perform routinely studies on the evolution of antimicrobial resistance in *Salmonella* strains of animal origin.

## Supplementary Files

Supplementary File 1 Supplementary (PDF, 72 KB)
