# Supplementary material for: Resistance Genes, Phage Types and Pulsed Field Gel Electrophoresis Pulsotypes in Salmonella enterica Strains from Laying Hen Farms in Southern Italy
Source: Int J Environ Res Public Health. 2013 Aug 6;10(8):3347–62. doi: 10.3390/ijerph10083347 (PMC3774442; doi:10.3390/ijerph10083347)
Supplement: Supplementary File 1 — Supplementary (PDF, 72 KB) [file ijerph-10-03347-s001.pdf]

Supplementary Information

Resistance Genes, Phage Types and Pulsed Field Gel Electrophoresis Pulsotypes in *Salmonella enterica* Strains from Laying Hen Farms in Southern Italy

Table S1. Primers used in this study.

Primer	5′-3′ sequence	Gene target	amplicon size (bp)	Reference
aadA1-F	TTTGATCAACGACCTTTTGGAAAC	*aadA1*	379	This study
aadA-R	GGACAACGTAAGCACTACATTTCG	*aadA1* and *aadA2*		
blaPSE-1-F	GGATTACAATGGCAATCAGCGCTTCC	bla_{PSE-1}	658	"
blaPSE-1-R	AATCGCATCATTTCGCTCTGCCATTG			
blaTEM1-F	TGAAGATCAGTTGGGTGCACGAGTGG	bla_{TEM-1}	700	"
blaTEM1-R	AGTTGCCTGACTCCCCGTCGTGTAGA			
floR-F	CTCCTTTCGACATCCTCGCTTCACTG	*floR*	636	"
floR-R	AGAAGACGAAGAAGGTGCCCATACCG			
intI1-F	CGAACCGAACAGGCTTATGTCCACTG	*intI1*	838	"
intI1-R	CATCGTCGTAGAGACGTCGGAATGG			
strA-F	TGGTTGCCTGTCAGAGGCGGAGAATC	*strA*	1466	"
strB-R	ATCGACGTCCAGCGCACGAGAGAATG	*strB*		
sul1-F	TCGGCATTCTGAATCTCACCGAGGAC	*sul1*	786	"
sul1-R	AAATTTCGCGAGGGTTTCCGAGAAGG			
sul2-F	GACAGTTTCTCCGATGGAGGCCGGTA	*sul2*	700	"
sul2-R	GTGTGCGGATGAAGTCAGCTCCACCT			
tetB-F	CAGGTTATCTTTGCTCCTTGGCTTGG	*tet*(B)	1014	"
tetB-R	TTGAGGGGTTAACATGAAGGTCATCG			
tetC-F	GGATATCGTCCATTCCGACAGCATCG	*tet*(C)	745	"
tetC-R	GATAATGGCCTGCTTCTCGCCGAAAC			
tetG-F	GAGCCGCAGTCGATTACACGATTATG	*tet*(G)	680	"
tetG-R	CAACAGAATCGGGAACACCATCCATC			
tnpATn3-F	ATAAGCAACGAACGTCCTGGCCTGCT	Tn*3 tnpA*	2544	"
tnpATn3-R	CACCAGTTCTGCCAGCGTGAAGGAAT			
tnpRTn3-F	GTGGAGCGGGCAATACTGAGCTGATG	Tn*3 tnpR*	361	"
tnpRTn3-R	AGGTGGAGGAAGGTGATGTCATTCTGG			
tnpAIS26-F	AATGTCGATCACTCCACGATTTACCG	IS*26 tnpA*	548	"
tnpAIS26-R	CCCAGGGGATCACCATAATAAAATGC			

Table S1. *Cont.*

Primer	5′-3′ sequence	Gene target	amplicon size (bp)	Reference
IS26-F	GGCACTGTTGCAAATAGTCGGTGGTG	IS*26* IR-*tnpA*	820	"
IS26-R	GGCACTGTTGCAAAGTTAGCGATGAG			
5CS-F	GCCTCGGGCATCCAAGCAGCAAGC	5'CS *attI1* end	variable	"
3CS-R	CTTGACCTGATAGTTTGGCTGTGAGCAA	3'CS		
tetA-F	GTAATTCTGAGCACTGTCGC	*tet*(A)	957	[1]
tetA-R	CTGCCTGGACAACATTGCTT			
U7-L12	ACACCTTGAGCAGGGCAAAG	*tdhF*	500	[2]
LJ-R1	AGTTCTAAAGGTTCGTAGTCG	*int*		
104-RJ	TGACGAGCTGAAGCGAATTG	S044	515	"
C9-L	AGCAAGTGTGCGTAATTTGG	*int2*		

Table S2. Distribution of *S.* Enteritidis and *S.* Typhimurium strains isolated from laying hen farms in 1998–2007.

**Year**	**Place**	**Samples**			**Inspections**		**Farms**		
		Positive (total)	***S.Enteritidis/ S. Typhimurium***	***Phage type (number)***	***Positive (total)***	***S.Enteritidis/ S. Typhimurium***	***Positive (total)***	***S.Enteritidis/ S. Typhimurium***	***Name***
1998	Apulia	2 (420)	2/0	PT (1); PT37 (1)	2 (42)	2/0	2 (14)	2/0	A, B
	Basilicata	0 (60)	0/0	-	0 (6)	0/0	0 (2)	0/0	-
1999	Apulia	3 (560)	0/3	DT193 (2); NT (1)	1 (56)	0/1	1 (14)	0/1	C
	Basilicata	1 (80)	0/1	DT193 (1)	1 (8)	0/1	1 (2)	0/1	D
2000	Apulia	5 (420)	0/5	DT193 (2); DT104 (1); U302 (1); DT208 (1)	5 (42)	0/5	5 (14)	0/5	C, E, F, G, H
	Basilicata	0 (60)	0/0	-	0 (6)	0/0	0 (2)	0/0	-
2001	Apulia	3 (560)	3/0	PT8 (3)	1 (56)	1/0	1 (14)	1/0	I
	Basilicata	0 (80)	0/0	-	0 (8)	0/0	0 (2)	0/0	-
2002	Apulia	0 (420)	0/0	-	0 (42)	0/0	0 (14)	0/0	-
	Basilicata	0 (60)	0/0	-	0/(6)	0/0	0 (2)	0/0	-
2003	Apulia	6 (560)	6/0	PT4 (1); PT14b (5)	3 (56)	3/0	2 (14)	2/0	J, K
	Basilicata	0 (80)	0/0	-	0 (8)	0/0	0 (2)	0/0	-
2004	Apulia	10 (420)	10/0	PT14b (7); NT (3)	3 (42)	3/0	2 (14)	2/0	C, L
	Basilicata	0 (60)	0/0	-	0 (6)	0/0	0 (2)	0/0	-
2005	Apulia	1 (560)	1/0	NT (1)	1 (56)	1/0	1 (14)	1/0	M
	Basilicata	0 (80)	0/0	-	0 (8)	0/0	0 (2)	0/0	-
2006	Apulia	7 (420)	6/1	PT14b (4); NT (2); DT104 (1)	4 (42)	3/1	4 (14)	3/1	E, L, N, O
	Basilicata	0 (60)	0/0	-	0 (6)	0/0	0 (2)	0/0	-
2007	Apulia	0 (560)	0/0	-	0 (56)	0/0	0 (14)	0/0	-
2007	Basilicata	1 (80)	0/1	DT104 (1)	1 (8)	0/1	1 (2)	0/1	P
Total		39 (5,600)	28/11	-	22 (560)	13/9	-	-	16

References

1. Iwanaga, M.; Toma, C.; Miyazato, T.; Insisiengmay, S.; Nakasone, N.; Ehara, M. Antibiotic resistance conferred by a class I integron and SXT constin in Vibrio cholerae O1 strains isolated in Laos. *Antimicrob. Agents Chemother.* **2004**, *48*, 2364–2369.
2. Boyd, D.; Peters, G.A.; Cloeckaert, A.; Boumedine, K.S.; Chaslus-Dancla, E.; Imberechts, H.; Mulvey, M.R. Complete nucleotide sequence of a 43-kilobase genomic island associated with the multidrug resistance region of Salmonella enterica serovar Typhimurium DT104 and its identification in phage type DT120 and serovar Agona. *J. Bacteriol.* **2001**, *183*, 5725–5732.

© 2013 by the authors; licensee MDPI, Basel, Switzerland. This article is an open access article distributed under the terms and conditions of the Creative Commons Attribution license (http://creativecommons.org/licenses/by/3.0/).
